# Severe Pediatric Snakebite With Coagulopathy and Compartment Syndrome: Conservative Management With Plasma Exchange

**DOI:** 10.1002/ccr3.73039

**Published:** 2026-06-25

**Authors:** Zain Mohammed Al Muqbel, Abdulrahman Al‐Majmuei, Ali Haider Ali, Naseem Yusuf Fahad, Reema Ajjawi, Amal Al Dailami, Afshan Lone, Manal Al Maskati, Nasser Mohamed Mansoor

**Affiliations:** ^1^ Salmaniya Medical Complex Manama Bahrain; ^2^ Royal College of Surgeons in Ireland – Medical University of Bahrain Busaiteen Bahrain; ^3^ Pediatric Emergency Salmaniya Medical Complex Manama Bahrain; ^4^ Emergency Medicine Salmaniya Medical Complex Manama Bahrain

**Keywords:** case report, compartment syndrome, disseminated intravascular coagulation (DIC), pediatric envenomation, snakebite, therapeutic plasma exchange, venom‐induced consumption coagulopathy (VICC)

## Abstract

Snakebite envenomation is a global public health concern, and hemotoxic bites can lead to severe coagulopathy, microangiopathic hemolytic anemia, and compartment syndrome. We describe a previously healthy 6‐year‐old boy who presented with progressive left lower limb swelling, discoloration, and bleeding from intravenous cannula sites following a snakebite sustained in rural Pakistan, consistent with severe hemotoxic envenomation. Despite antivenom and transfusion support, he developed persistent venom‐induced consumption coagulopathy with hypofibrinogenemia, markedly prolonged clotting times, thrombocytopenia, and features of microangiopathic hemolytic anemia. Severe limb swelling raised concern for compartment syndrome; however, fasciotomy was deferred due to the high risk of bleeding in the setting of uncontrolled coagulopathy. Transferred to Bahrain, he underwent five sessions of therapeutic plasma exchange, initiated due to ongoing clinical and laboratory deterioration, resulting in stabilization of hematologic parameters, resolution of limb swelling, and preservation of limb function. This case highlights the role of plasma exchange in children unresponsive to conventional therapy and demonstrates that conservative management of suspected compartment syndrome may be feasible when surgical intervention carries significant risk, underscoring the importance of early recognition and multidisciplinary care in complex pediatric envenomation.

## Key Clinical Message

Therapeutic plasma exchange can be a lifesaving intervention in pediatric snakebite with refractory coagulopathy and compartment syndrome when standard therapies fail.

## Graphical abstract

Therapeutic plasma exchange was successfully used in a child with severe snakebite envenomation complicated by refractory venom‐induced consumptive coagulopathy and suspected compartment syndrome, leading to full recovery and avoidance of surgical intervention.

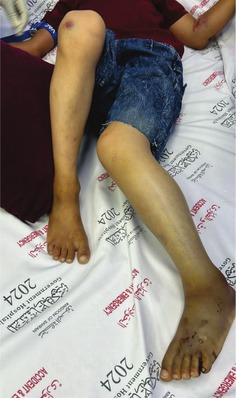

## 1 Introduction

Snakebite envenomation and associated mortality represent a significant global public health problem, disproportionately affecting rural populations in tropical regions, particularly in Southeast Asia and sub‐Saharan Africa [1]. The World Health Organization (WHO) classifies snakebite envenomation as a neglected tropical disease, with an estimated 5.4 million bites and 2.7 million envenomations annually [2].

In South Asia, including Pakistan, where this envenomation occurred, medically important snakes predominantly belong to the so‐called “big four”: *Daboia russelii* (Russell's viper), *Echis carinatus* (saw‐scaled viper), *Naja naja* (Indian cobra), and *Bungarus caeruleus* (common krait) [3]. These species are responsible for the majority of clinically significant envenomations in the region. Viperidae species, particularly Russell's viper and saw‐scaled viper, are most commonly associated with hemotoxic envenomation, characterized by venom‐induced consumption coagulopathy, bleeding diathesis, and local tissue injury [4]. In contrast, elapid snakes such as cobras and kraits predominantly cause neurotoxic effects, including progressive paralysis and respiratory compromise [5]. The clinical features observed in this patient, including progressive limb swelling and severe coagulopathy, are therefore most consistent with viper envenomation.

Pediatric and elderly patients are particularly vulnerable, as envenomation can rapidly cause local or systemic complications, sometimes fatal within 6 h [5]. Children are at increased risk due to their smaller total blood volume, which amplifies the toxic effects of a given venom dose [5].

Hemotoxic snake venoms commonly cause venom‐induced consumption coagulopathy (VICC), characterized by hypofibrinogenemia, elevated D‐dimer levels, and prolonged clotting times [6]. In severe cases, this may resemble or overlap with disseminated intravascular coagulation (DIC), although the underlying mechanisms differ [6]. Clinical progression can be further complicated by microangiopathic hemolytic anemia (MAHA), thrombocytopenia, and although rare, life‐threatening compartment syndrome [5].

The cornerstone of management is prompt administration of species‐specific antivenom [5]. However, some patients develop persistent coagulopathy despite adequate antivenom therapy [4]. In such cases, supportive measures including blood product transfusion and, in selected cases, prothrombin complex concentrates, recombinant factor VII, and therapeutic plasma exchange may be considered [5, 7]. Evidence supporting therapeutic plasma exchange in pediatric snakebite remains limited, with its use largely guided by clinical judgment in cases of refractory envenomation [8].

We report a case of a 6‐year‐old boy who developed refractory coagulopathy, MAHA, and compartment syndrome following snakebite envenomation. He was successfully managed with therapeutic plasma exchange. This case highlights the complexity of managing severe hemotoxic envenomation and the potential role of plasma exchange as an adjunctive therapy in carefully selected pediatric patients.

## 2 Case History/Examination

A previously healthy 6‐year‐old boy presented to the Emergency Department in Bahrain on 27 August 2025 with painful swelling, discoloration, and restricted movement of his left lower limb. These symptoms developed 4 days after he sustained a bite to the dorsum of his left foot while putting on his slipper at home in rural Pakistan. Although the snake was not identified, the clinical history of a bite sustained in a rural setting, together with puncture marks, progressive local swelling, and subsequent development of coagulopathy, strongly supported the diagnosis of snake envenomation, most consistent with a hemotoxic species.

Initial management at a local hospital included 20 mL of antivenom, a single dose of dexamethasone, anti‐inflammatory therapy, and placement of an intravenous cannula for fluid administration, medication delivery, and emergency access. He was discharged with instructions to undergo coagulation testing at a city hospital. He later received a second dose of antivenom at a tertiary hospital in Islamabad, along with oral antibiotics (amoxicillin/clavulanic acid and linezolid), before being cleared to travel to Bahrain. Upon arrival, his father reported oozing from the cannula site and worsening pain and swelling, prompting presentation to the Emergency Department.

**FIGURE 1:**
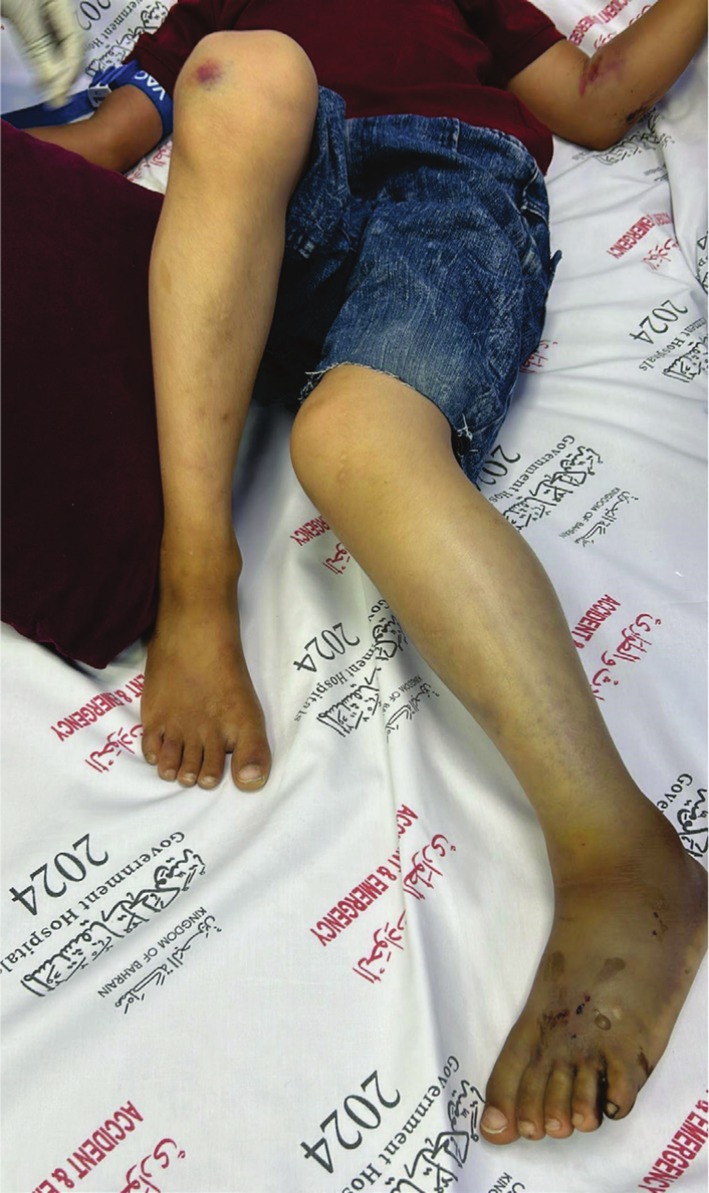
Left lower limb showing swelling, discoloration, and the bite site following envenomation.

The patient was drowsy but easily arousable, with a Glasgow Coma Scale of 15 out of 15, breathing comfortably on room air and connected to a cardiac monitor. Vital signs were stable, and general examination was unremarkable except for the affected limb. The left lower limb showed swelling extending to the thigh, with a tense dorsum, grayish‐blue discoloration, a blackish bite mark, and a small blister (Figure 1). Capillary refill was delayed at 5 s, and the leg was tender with pain on passive movement, though distal pulses were palpable and preserved. These findings, particularly pain out of proportion to examination and tense swelling, raised concern for evolving acute compartment syndrome. The right upper limb had a hematoma at the previous intravenous cannula site with mild localized edema and tenderness. Other systemic examinations were unremarkable. At initial presentation, the patient's clinical picture was predominantly consistent with local envenoming, with systemic features developing subsequently.

The patient developed severe hematologic toxicity after snake envenomation, characterized by venom‐induced consumption coagulopathy with rapid depletion of clotting factors, microangiopathic hemolytic anemia evidenced by schistocytes at 2.2%, anisocytosis, microcytosis, hemoglobin drop from 13.4 g per decilitre to 6.5 g per decilitre, elevated lactate dehydrogenase from 249 to 909 units per liter and creatine kinase from 261 to 631 units per liter, and indirect bilirubin of 44.5 μmol per liter, and progressive thrombocytopenia from 361 × 10^9^ per liter to 52 × 10^9^ per liter, consistent with consumptive coagulopathy. Platelet transfusions were administered but counts remained unstable due to ongoing venom‐induced consumption. Coagulation studies, including prothrombin time, activated partial thromboplastin time, international normalized ratio, and fibrinogen, were repeatedly non‐reportable, indicating severe coagulopathy and features of disseminated intravascular coagulation. C‐reactive protein rose from < 4 to 176 mg per liter, while procalcitonin was mildly elevated from 0.3 to 1.78 nanograms per milliliter; cultures were negative.

Imaging included a Doppler ultrasound showing cellulitis and soft tissue edema without deep vein thrombosis. Doppler assessment demonstrated preserved distal arterial flow, helping to exclude vascular occlusion as a cause of the limb findings. The diagnosis of compartment syndrome was made clinically, based on progressive tense swelling and pain on passive movement. X‐ray excluded foreign bodies, and echocardiography revealed mild pulmonary hypertension with otherwise normal cardiac structure. Given the high clinical suspicion of acute compartment syndrome, surgical fasciotomy was considered; however, this was deferred due to severe coagulopathy and the significant risk of uncontrolled bleeding. The patient was therefore managed conservatively with close neurovascular monitoring, limb elevation, and aggressive correction of coagulopathy to prevent limb loss while awaiting stabilization. See Table 1 for a summary of the clinical course and management, and Table 2 for the hospitalization timeline and laboratory correlation.

**TABLE 1:** Chronological summary of the patient's clinical course and management.

Time period	Clinical status	Key findings	Interventions
Day 0–1	Initial presentation and stabilization	Limb swelling and pain; coagulopathy; concern for compartment syndrome	Second dose of antivenom (ED and PICU); IV fluids; limb elevation; IV paracetamol; wound care; proton pump inhibitor; IV amoxicillin‐clavulanate; fresh frozen plasma and cryoprecipitate; orthopedic consultation; fasciotomy deferred due to coagulopathy; baseline labs and Doppler imaging to exclude deep vein thrombosis
Day 2–10	Persistent severe disease	Ongoing MAHA and refractory VICC; tense swelling, bullae, and discoloration of left foot; swelling and tenderness of right hand (concern for Volkmann contracture)	Therapeutic plasma exchange (5 sessions, 1:1 plasma replacement with fibrinogen); blood products as needed; prothrombin complex concentrate and recombinant factor VIIa; escalation to cefepime, meropenem, and vancomycin; sterile bullae aspiration; limb elevation; methylprednisolone 1 mg/kg following plasmapheresis; imaging confirming cellulitis without deep vein thrombosis or foreign bodies
Day 11–15	Recovery phase	Stabilization of hemoglobin, platelet count, and coagulation profile; reduced swelling and improved passive movement; preserved pulses; no necrosis	Continued supportive care including oral nutrition, analgesia, limb elevation, and sterile wound dressings
Discharge & follow‐up	Clinical stability at discharge	No progression to limb‐threatening complications	Topical povidone‐iodine for wound care; aspirin 81 mg daily; follow‐up with pediatric hematology in 1 month for complete blood count and coagulation assessment; safety‐netting advice provided

**TABLE 2:** Timeline of hospitalization with laboratory and clinical correlation.

Date	Hb (g/dL)	Platelets (×10^9^/L)	LDH (U/L)	CK (U/L)	PT/INR/APTT/fibrinogen	CRP (mg/L)	Na (mmol/L)	K (mmol/L)	Urea (mmol/L)	Creatinine (μmol/L)
Reference range	11.5–15.5	150–450	140–280	30–200	PT 11–14 s/INR 0.8–1.2/APTT 25–35 s/fibrinogen 200–400 mg/dL	< 5	136–145	3.5–5.1	1.8–6.4	20–44
Preadmission	13.4	361	—	—	—	< 4	—	—	—	—
Day 0 (arrival)	10.7	205	336	441	Not deranged	< 4	137	4.0	4.6	25
Day 2	9.1	135	574	631	Not deranged	37	138	4.0	4.4	28
Day 3	6.5	52	876	571	Not deranged	76	137	3.9	4.0	20
Day 5	8.5	116	904	424	Not deranged	176	134	3.1	6.0	23
Day 9	9.4	507	629	230	PT 15.3 s/INR 1.37/fibrinogen 49.1 mg/dL/D‐dimer 30.6 mg/L	—	137	5.6	10.0	19
Day 15 (discharge)	9.5	181	569	230	PT 11.5 s/INR 1.02/fibrinogen 153.9 mg/dL/D‐dimer 1.10 mg/L	—	138	4.0	4.0	23

Renal involvement was also observed during the clinical course. Blood urea was elevated at 10.0 mmol/L (reference range 1.8–6.4), while serum creatinine was 19 μmol/L (reference range 20–44). Urine output remained preserved throughout admission, with no documented oliguria. These findings did not meet the criteria for acute kidney injury. Serum electrolyte analysis demonstrated sodium of 137 mmol/L (reference range 136–145), potassium of 5.6 mmol/L (reference range 3.5–5.1), chloride of 102 mmol/L (reference range 98–107), and bicarbonate of 25 mmol/L (reference range 20–31), indicating mild hyperkalaemia without metabolic acidosis.

The constellation of venom‐induced consumption coagulopathy, microangiopathic haemolytic anemia, and thrombocytopenia is consistent with severe hemotoxic envenomation. Despite laboratory evidence of haemolysis, the absence of acute kidney injury makes thrombotic microangiopathy unlikely in this case.

## 3 Differential Diagnosis, Investigations and Treatment

The differential diagnosis included severe hemotoxic snake envenomation with venom‐induced consumption coagulopathy, disseminated intravascular coagulation, compartment syndrome, cellulitis, deep vein thrombosis, vascular compromise, and snakebite‐associated thrombotic microangiopathy.

The patient developed severe hematologic toxicity after snake envenomation, characterized by venom‐induced consumption coagulopathy with rapid depletion of clotting factors, microangiopathic hemolytic anemia, and progressive thrombocytopenia. Platelet transfusions were administered but counts remained unstable due to ongoing venom‐induced consumption. Coagulation studies were repeatedly non‐reportable, indicating severe coagulopathy and features of disseminated intravascular coagulation.

Imaging included Doppler ultrasonography demonstrating cellulitis and soft tissue edema without deep vein thrombosis, while distal arterial flow remained preserved. X‐ray excluded retained foreign bodies, and echocardiography revealed mild pulmonary hypertension with otherwise normal cardiac structure.

Given the high clinical suspicion of acute compartment syndrome, surgical fasciotomy was considered but deferred because of severe coagulopathy and the substantial risk of uncontrolled bleeding. The patient was managed conservatively with close neurovascular monitoring, limb elevation, blood product support, antivenom therapy, and five sessions of therapeutic plasma exchange. Renal function remained preserved throughout admission, making snakebite‐associated thrombotic microangiopathy unlikely despite evidence of hemolysis and thrombocytopenia.

## 4 Outcome and Follow‐Up

Following therapeutic plasma exchange and ongoing supportive management, the patient's hematologic parameters gradually stabilized, with improvement in platelet count, fibrinogen level, and coagulation profile. Limb swelling progressively resolved, passive movement improved, and distal perfusion remained preserved without development of tissue necrosis or permanent functional impairment.

The patient was discharged clinically stable on topical povidone‐iodine wound care and aspirin 81 mg daily, with planned pediatric hematology follow‐up. At follow‐up, no progression to limb‐threatening complications was observed, limb function was preserved, and laboratory parameters continued to improve.

## 5 Discussion

Snake envenomation can result in a wide spectrum of local and systemic complications, with hemotoxic bites frequently causing venom‐induced consumption coagulopathy and, in severe cases, microangiopathic hemolytic anemia [5]. Although compartment syndrome is an uncommon consequence, it represents a critical complication due to the risk of irreversible tissue ischemia and limb loss if not promptly recognized and managed [9].

In this case, the clinical picture is most consistent with hemotoxic viper envenomation based on a syndromic approach. The combination of progressive local swelling, hypofibrinogenemia, thrombocytopenia, and hemolysis strongly supports Viperidae envenomation, particularly species such as Russell's viper or saw‐scaled viper [5]. The absence of neurotoxic features further excludes elapid envenomation, reinforcing the value of clinical pattern recognition when species identification is not possible [5].

The key challenge was managing suspected compartment syndrome in the setting of severe coagulopathy, where the risk of uncontrolled bleeding made immediate fasciotomy unsafe. At the same time, delaying intervention carried the risk of irreversible tissue injury. This required a carefully balanced approach, prioritizing correction of coagulopathy while maintaining close clinical monitoring to guide the timing of any surgical intervention.

Importantly, this case demonstrates that conservative management of suspected compartment syndrome may be a viable strategy in carefully selected patients when surgery is contraindicated. Close monitoring, limb elevation, and aggressive correction of coagulopathy allowed preservation of limb function without surgical intervention. This challenges the conventional assumption that all cases of compartment syndrome require immediate fasciotomy, suggesting that clinical context and reversibility of the underlying process must be considered.

The role of therapeutic plasma exchange in this case is particularly noteworthy. However, there is currently no established evidence supporting its use as a direct treatment for compartment syndrome. The available literature instead describes its role in severe snake envenomation complicated by refractory venom‐induced consumption coagulopathy or snakebite‐associated thrombotic microangiopathy, where it may be lifesaving in the presence of acute kidney injury [5, 8]. In this patient, despite marked hemolysis and thrombocytopenia, there was no evidence of renal impairment, making thrombotic microangiopathy unlikely. Therapeutic plasma exchange was therefore initiated to address persistent hematologic derangement rather than local tissue pathology. Any apparent benefit to the threatened limb was likely indirect, mediated by reductions in circulating venom‐related factors and correction of coagulopathy, which may have limited ongoing tissue injury and enabled safe avoidance of high‐risk surgical intervention [10, 11].

Venom‐induced thrombotic microangiopathy has been increasingly recognized as a complication of severe envenomation, characterized by hemolysis, thrombocytopenia, and acute kidney injury. Although plasma exchange has shown benefit in such cases, the absence of renal impairment in this patient argues against this diagnosis [11]. This distinction is important, as it suggests that plasma exchange may have utility beyond classical thrombotic microangiopathy, particularly in refractory coagulopathy without overt renal involvement.

This case also reinforces the importance of early and appropriate antivenom administration. Delayed or insufficient treatment can allow progression of both local and systemic toxicity, increasing the risk of complications such as compartment syndrome and persistent coagulopathy. In resource‐limited settings, where delays are more common, outcomes are often worse, further emphasizing the need for timely intervention and adaptable management strategies [12, 13, 14, 15].

In practice, snakebite management is not linear but requires continuous reassessment as complications evolve. The overlap between hematologic toxicity and surgical pathology necessitates close collaboration between specialties, particularly in pediatric patients, where disease progression may be rapid and less predictable.

## 6 Conclusion

This case highlights the challenge of managing suspected compartment syndrome in pediatric hemotoxic envenomation complicated by severe coagulopathy, where immediate fasciotomy may be unsafe. It demonstrates that a carefully monitored, conservative approach with correction of coagulopathy can preserve limb function. It also supports a potential adjunctive role for therapeutic plasma exchange in refractory cases, even in the absence of thrombotic microangiopathy. These findings emphasize the need for flexible, multidisciplinary decision‐making in complex envenomation.

## Author Contributions

**Zain Mohammed Al Muqbel:** conceptualization, data curation, formal analysis, funding acquisition, investigation, methodology, project administration, resources, supervision, validation, visualization, writing – original draft. **Abdulrahman Al‐Majmuei:** conceptualization, data curation, formal analysis, funding acquisition, investigation, methodology, resources, software, supervision, validation, visualization, writing – review and editing. **Ali Haider Ali:** conceptualization, data curation, formal analysis, funding acquisition, investigation, methodology, project administration, resources, software, supervision, validation, visualization, writing – original draft, writing – review and editing. **Naseem Yusuf Fahad:** conceptualization, data curation, formal analysis, funding acquisition, investigation, methodology, project administration, resources, software, supervision, validation, visualization, writing – original draft, writing – review and editing. **Reema Ajjawi:** conceptualization, data curation, formal analysis, funding acquisition, methodology, resources, supervision, validation, visualization, writing – review and editing. **Amal Al Dailami:** conceptualization, data curation, formal analysis, funding acquisition, investigation, methodology, project administration, resources, software, supervision, validation, visualization, writing – original draft. **Manal Al Maskati:** conceptualization, data curation, formal analysis, funding acquisition, investigation, methodology, project administration, validation. **Afshan Lone:** conceptualization, data curation, formal analysis, funding acquisition, methodology, resources, supervision, validation, visualization. **Nasser Mohamed Mansoor:** conceptualization, data curation, formal analysis, funding acquisition, investigation, methodology, project administration, resources, software, supervision, validation, visualization.

## Funding

The authors have nothing to report.

## Ethics Statement

The authors have nothing to report.

## Consent

Written informed consent was obtained from the patient's parents for publication of this case report and accompanying images. The authors confirm that the parents understood the nature of the publication and agreed to the inclusion of clinical details and photographs.

## Conflicts of Interest

The authors declare no conflicts of interest.

## Data Availability

All data relevant to this case report are included within the manuscript. No additional datasets were generated or analyzed during the current study.
